# Brazilian Green Propolis Improves Antioxidant Function in Patients with Type 2 Diabetes Mellitus

**DOI:** 10.3390/ijerph13050498

**Published:** 2016-05-13

**Authors:** Liting Zhao, Lingling Pu, Jingyu Wei, Jinghua Li, Jianquan Wu, Zhonghao Xin, Weina Gao, Changjiang Guo

**Affiliations:** 1 Department of Nutrition, Tianjin Institute of Health and Environmental Medicine, Tianjin 300050, China; zlt_medic@126.com (L.Z.); pulingling@163.com (L.P.); wjy55333@126.com (J.W.); xinzhonghaojk@163.com (Z.X.); 2 Department of Nutrition, Pingjin Hospital, Tianjin 300162, China; 3 Department of Endocrinology, Pingjin Hospital, Tianjin 300162, China; lijingh1020@163.com; 4 Department of Health Education, Tianjin Institute of Health Education, Tianjin 300011, China; wupp17@163.com

**Keywords:** Brazilian green propolis, type 2 diabetes mellitus, antioxidant function

## Abstract

Propolis contains a variety of bioactive components and possesses many biological properties. This study was designed to evaluate potential effects of Brazilian green propolis on glucose metabolism and antioxidant function in patients with type 2 diabetes mellitus (T2DM). In the 18-week randomized controlled study, enrolled patients with T2DM were randomly assigned to Brazilian green propolis group (900 mg/day) (*n* = 32) and control group (*n* = 33). At the end of the study, no significant difference was found in serum glucose, glycosylated hemoglobin, insulin, aldose reductase or adiponectin between the two groups. However, serum GSH and total polyphenols were significantly increased, and serum carbonyls and lactate dehydrogenase activity were significantly reduced in the Brazilian green propolis group. Serum TNF-α was significantly decreased, whereas serum IL-1β and IL-6 were significantly increased in the Brazilian green propolis group. It is concluded that Brazilian green propolis is effective in improving antioxidant function in T2DM patients.

## 1. Introduction

Diabetes mellitus (DM) is a chronic disease and characterized by insufficient insulin activity. DM causes multiple metabolic disorders, which are manifested by hyperglycemia and several complications such as hypoglycemia, cardiovascular disease, nerve damage, kidney failure, limb amputation and vision problems [1]. Type 2 diabetes mellitus (T2DM) is the most common type of DM, in which insulin resistance develops and leads to a decreased response of the peripheral tissues to insulin activity. Currently, it is estimated that about 382 million people all over the world are diagnosed with T2DM [2]. In China, the number of patients with T2DM has been increasing dramatically in recent years and as many as 98.4 million cases were reported in 2013 [2].

Propolis is a resinous material produced by bees using the collected exudates and buds of plants in combination with beeswax and enzymes. Propolis is well known for its biological properties, including antioxidant, antiviral, antibacterial, antifungal, antiatherogenic, and antiproliferative activities [3,4,5,6,7,8]. Propolis is rich in active components, such as phenolic acids, flavonoids, terpenes, beeswax as well as proteins, sugars, vitamins and elements and has been used for folk medicine in many countries [9,10,11]. Previously, propolis was demonstrated to be potential in improving glucose metabolism and antioxidant function in diabetic rats, indicating that propolis may be useful in the prevention and treatment of DM [12]. However, Fukuda *et al.* [13] found that there was no evident difference between propolis group and placebo group in the changes of homeostasis model assessment for insulin resistance, haemoglobinA1C, fasting blood glucose or serum insulin level in T2DM patients after an 8-week intervention. Thereby, further clinical studies should be performed to verify whether propolis is useful in the treatment of T2DM.

In the present study, we hypothesized that propolis is effective in the treatment of T2DM. To test this hypothesis, an 18-week randomized clinic trial was performed to investigate the effects of Brazilian green propolis on glucose metabolism and antioxidant function in patients with T2DM.

## 2. Materials and Methods

### 2.1. Study Subjects

From May to September in 2013, a total of 70 T2DM patients, aged 35–78 years, were recruited from the Department of Endocrinology, Pingjin Hospital, Tianjin, China. Written informed consent was signed by all patients. T2DM was diagnosed in accordance with the criteria of the American Diabetes Mellitus Association. The candidate patients were informed of the study aims and procedures. The patients with any of the following conditions were excluded from the study: (1) substance abuse including smoking or heavy drinking; (2) any kinds of allergies; (3) use of medications including insulin, hormonal contraceptives and other kinds of functional foods or health products; (4) serious cardiovascular, hematological, renal, respiratory, gastrointestinal, endocrine or central nervous system diseases, psychiatric disorders, active cancers, acute inflammation or infection based on medical history and physical or laboratory examinations obtained at recruitment; (5) women in pregnancy or lactation. All subjects gave their informed consent for inclusion before they participated in the study. The study was conducted in accordance with the Declaration of Helsinki, and the protocol was approved by the Ethics Committee of Tianjin Institute of Health and Environmental Medicine (TIHE-TY-20130326).

### 2.2. Study Design

Brazilian green propolis was selected for this study because it is rich in phenolics [9]. It was purified by ethanol extraction. After being mixed with soybean oil and glycerin, it was encapsulated by the By-Health Co., Ltd. (Guangzhou, China). The patients enrolled in this study were randomly divided into the control and Brazilian green propolis groups based on their fasting serum glucose levels. At baseline, body height and weight, waist and hip circumferences were measured. Body mass index (BMI) was calculated as body weight (kg)/(body height (m))^2^. Waist to hip ratio (WHR) was determined as waist (cm)/hip (cm). Fasting blood samples were collected and biochemical parameters related to glucose metabolism and antioxidant function were assayed. Patients in the Brazilian green propolis group took Brazilian propolis in capsules at the dose of 900 mg daily for 18 weeks. The dose was extrapolated from animal experiments reported previously [14,15]. All treatments, diabetic diets or exercise regimens at baseline were continued and without any change throughout the intervention period. At the end of the study, fasting blood samples were collected and biochemical parameters were analyzed again accordingly.

### 2.3. Dietary Survey

During the last week of the study, a 5-day dietary survey was conducted using a 24 h recall method by experienced interviewers in order to make comparison in dietary intake of energy and nutrients between the two groups. After the survey, the intakes of energy and nutrients were calculated based on food intakes and Chinese Food Composition, which was compiled by the Institute of Nutrition and Food Safety, Chinese Center for Disease Control and Prevention [16].

### 2.4. Measurement of Blood Biochemical Parameters

Fasting blood samples from the antecubital vein were collected at the beginning and the end of the intervention. Vacuum tubes were used to obtain blood samples. Serum glucose was assayed by a commercial kit purchased from BioSino Bio-technology and Science Inc. (Beijing, China). Serum insulin was determined by a chemiluminescent enzyme immunoassay kit purchased from Huanri Inc. (Shandong, China). Serum glycosylated hemoglobin was measured by a glycosylated hemoglobin analyzer (Bio-Rad Laboratories, Hercules, CA, USA) based on a high performance liquid chromatography method. Serum aldose reductase and adiponectin were analyzed by two commercial enzyme-linked immunosorbent assay (ELISA) kits obtained from BD Biosciences (Lake Franklin, NJ, USA).

The ferric-reducing antioxidant power (FRAP) assay described by Benzie and Strain [17] was used to analyze serum antioxidant capacity. The activities of serum superoxide dismutase (SOD), glutathione peroxidase (GSH-Px) were measured by two commercial kits purchased from Jiancheng Bioengineering Institute (Nanjing, China). Serum reduced glutathione (GSH) was assayed spectrophotometrically by the reaction of 5,5’-dithiobis-2-nitrobenzoic acid with thiols. The reagent kit was purchased from Jiancheng Bioengineering Institute. Serum total polyphenols was determined spectrophotometrically by Folin-Ciocalteu method as described previously [18]. A BioTek uQuant spectrophotometer (BioTek Instruments Inc., Winooski, VT, USA) was used.

Serum malondialdehyde (MDA) equivalents were assayed spectrophotometrically by the reaction with thiobarbituric acid [19]. Serum oxidized low density lipoprotein (ox-LDL) was measured by a commercial ELISA kits from BD Biosciences. Serum carbonyls were detected by the reaction with 2,4-dinitrophenylhydrazine as reported by Levine *et al.* [20] and the reagent kit was purchased from Jiancheng Bioengineering Institute. Lactate dehydrogenase (LDH) was measured spectrophotometrically by an assay kit purchased from Jiancheng Bioengineering Institute. Serum interleukin-1β (IL-1β), interleukin-6 (IL-6) and tumor necrosis-factor α (TNF-α) were measured by commercial ELISA kits from BD Biosciences.

### 2.5. Statistical Analysis

Statistical analysis was performed using the SPSS 10.0 software (SPSS Inc., Chicago, IL, USA). Data are expressed as means ± standard deviation and checked for normality using Kolmogorov-Smirnov test before subjected to further analysis. When the data are in normality, Student’s *t*-test was used to analyze the difference between the two groups. Otherwise, Mann-Whitney rank sum test was performed. Statistic significances were accepted at *p* < 0.05.

## 3. Results

### 3.1. General Characteristics of the Patients at Baseline

Seventy patients were qualified to participate in this study at the beginning. Sixty-five patients completed the intervention. Two patients from the Brazilian green propolis group withdrew from the study because of allergies. One patient from the control group dropped out due to heart attack. In addition, two patients from the control group left the study for personal reasons. Their data were excluded from the final statistical analysis. As shown in Table 1, there was no significant difference between the two groups in body weight, BMI and serum parameters related to glucose metabolism and antioxidant function.

### 3.2. Dietary Intake of Energy and Nutrients

There was no significant difference in dietary intake of energy and most nutrients between the two groups except ascorbic acid and calcium. The patients in the Brazilian green propolis group were significantly lower in dietary intake of ascorbic acid and calcium than those in the control group by 26.2% and 27.5%, respectively (Table 2).

### 3.3. Effects of Brazilian Green Propolis on Glucose Metabolism

No significant difference was found in serum glucose, glycosylated hemoglobin, insulin, aldose reductase and adiponectin between the two groups at the end of the study (Table 3), indicating that Brazilian green propolis exerts no significant impact on glucose metabolism in T2DM patients.

### 3.4. Effects of Brazilian Green Propolis on Antioxidant Function and Cytokines

After administration of Brazilian green propolis, serum GSH and total polyphenols were significantly increased in comparison with the control. Meanwhile, serum carbonyls and LDH were significantly reduced, indicating that Brazilian green propolis is effective in improving some markers of antioxidant function. However, no significant difference was noted for serum FRAP, SOD, GSH-Px, MDA or Ox-LDL between the two groups. Interestingly, serum TNF-α was decreased significantly whereas serum IL-1β and IL-6 were increased significantly in the Brazilian green propolis group than in the control group (Table 4).

## 4. Discussion

Oxidative stress is defined as an imbalance between reactive oxygen species (ROS) production and neutralization and considered one of critical factors in the pathogenesis and development of T2DM [21,22,23]. Many studies demonstrated that T2DM patients suffered from increased ROS production and oxidative damage, indicating that antioxidant function is impaired to some extent [24,25]. Hyperglycemia condition can induce oxidative stress in T2DM by several mechanisms, such as glucose autoxidation, polyol pathway and formation of glycosylated products [26,27,28,29]. In addition, dislipidemia and low-grade inflammation also contribute significantly to the oxidative stress in T2DM patients [25,30]. Several studies revealed that antioxidant supplementations, such as natural polyphenols, were effective in the protection against oxidative stress in T2DM animals and patients [26,31,32]. Propolis is rich in polyphenols and presents strong antioxidant activity. Currently, more than 300 chemical components belonging to the phenolics and flavonoids and terpenes have been isolated from the propolis [10,11,33]. Previously, we found that Brazilian green propolis contained as much as 189.12 mg/g of total polyphenols [33]. El-Awady *et al.* [34] reported that propolis not only possessed abundant bioactive constituents and a variety of biological activities, but also protected against high glucose-induced vascular dysfunction by reducing oxidative stress. An animal experiment showed that the protective effect of propolis on hepatorenal function was partially attributed to its antioxidant activity [15]. We also observed that Brazilian green propolis was effective in improving antioxidant function in diabetic rats [14]. In the present study, we found for the first time that Brazilian green propolis could significantly increase serum GSH, an important antioxidant in the body, and decrease serum carbonyls, a marker for oxidized proteins, in T2DM patients after 18-week administration. Interestingly, serum total polyphenols were significantly increased simultaneously, indicating a possibility that the polyphenols contained in the Brazilian green propolis are bioavailable and can function as antioxidants *in vivo* after absorption. However, serum MDA equivalents and ox-LDL, two important markers for lipid peroxidation, were not reduced after the treatment of Brazilian green propolis. It is not surprising because most polyphenols are water soluble in nature and may not act as an important antioxidant in the lipid soluble phases.

Several animal studies showed that propolis displayed a remarkable effect on glucose homeostasis. Al-Hariri *et al.* and Zhu *et al.* [35,36] demonstrated that propolis could improve blood glucose level and function of the pancreatic islets in streptozotocin-induced diabetic rats. Li *et al.* [15] found that propolis could effectively control blood glucose and increase insulin sensitivity in T2DM rats. Hu *et al.* [37] reported a similar result in rats with DM. However, it is regrettable that we did not confirm the hypoglycemic effects of propolis in T2DM patients in the present study. A consistent result was also reported previously by Fukuda *et al.* [13], in which they found that the 8-week administration of Brazilian green propolis failed to improve glucose metabolism significantly, though it was effective in the prevention against hyperuricemia and dysfunction of renal glomerular filtrating function in T2DM patients. Currently, we do not have an explanation for this discrepancy between animal experiments and clinic trials. Further studies with well controlled design are necessary to be performed in order to verify the potential role of Brazilian green propolis in improving glucose metabolism in T2DM patients.

IL-1β, IL-6 and TNF-α are considered pro-inflammatory cytokines because they play an important role in chronic inflammation [38]. It has been shown that T2DM patients had higher levels of inflammatory cytokines, which significantly contributes to the chronic inflammation and oxidative stress in T2DM [39]. Recently, it was found that IL-6 was effective in suppressing inflammation in several animal models and the mechanisms are possibly associated with inhibition of IL-1β and TNF-α production, as well as the induction of IL-1Ra synthesis and release of soluble TNF-α receptors in the circulation [40,41]. Thereby, IL-6 is also considered an anti-inflammatory cytokine in certain circumstances [40]. In this study, we observed that serum TNF-α was significantly decreased after the treatment of Brazilian green propolis in T2DM patients. In contrast, serum IL-1β and IL-6 were significantly increased. It is explainable since propolis, as well as some of its components, was active in stimulating the secretion of several cytokines, including IL-1β *in vitro* [42]. However, the pro-inflammatory effects resulted from increasing IL-1β production could be possibly counteracted by the anti-inflammatory effects of IL-6, which is also significantly increased in this study. Therefore, the combined action of Brazilian green propolis on chronic inflammation is favorable in T2DM patients. This is supported partially by decreased LDH activity in the serum as measured in this study. LDH, an enzyme involved in L-lactate metabolism, is crucial to the pathophysiology and therapy of T2DM [43]. Under normal circumstances, the release of cellular LDH into the circulation is limited. However, a large number of cells could be damaged in inflammation, which is accompanied by a significant rise in serum LDH activity [44,45]. The decline in serum LDH activity in this study suggests that administration of Brazilian green propolis is protective against cellular injury in T2DM.

## 5. Conclusions

Taken together, this study demonstrates that Brazilian green propolis is effective in improving antioxidant function in T2DM patients, which is associated with increased serum GSH, polyphenols and anti-inflammatory cytokines. The underlying mechanisms need to be further explored. However, we are aware that this study has several limitations. First, we did not provide a placebo for the control group, which might have had some psychological impact on subjects in that group. Second, we did not measure serum contents of ascorbic acid, tocopherol and other antioxidants. Although the dietary survey results showed that the intakes of most nutrients were similar between the two groups, the intake of ascorbic acid and calcium was significantly lower in the Brazilian green propolis group. This may compromise partially the antioxidant and anti-inflammatory effects of Brazilian green propolis. Finally, Brazilian green propolis was administrated only at a high dose level in this study. Thereby, the adequate doses of Brazilian green propolis still need to be further investigated before clinic applications are conducted at a large scale.

## Figures and Tables

**Table 1:** General characteristics of the subjects at baseline.

Parameter	Control	Brazilian Green Propolis
Male	14	18
Female	18	15
Total	32	33
Age (year)	60.8 ± 8.6	59.5 ± 8.0
Body height (cm)	164.8 ± 8.2	166.1 ± 7.9
Body weight (kg)	73.9 ± 9.4	71.5 ± 10.3
WHR	0.94 ± 0.06	0.92 ± 0.05
BMI (kg/m^2^)	27.2 ± 3.4	25.8 ± 2.4
Glucose (mmol/L)	8.5 ± 2.6	9.0 ± 3.0
Glycosylated hemoglobin (%)	7.9 ± 1.5	8.2 ± 1.5
Insulin (uIU/mL)	12.2 ± 3.8	13.8 ± 3.3
FRAP (mmol/L)	0.68 ± 0.14	0.71 ± 0.11
SOD (U/mL)	108.3 ± 26.1	110.1 ± 18.9
GSH-Px (U/L)	193.7 ± 71.2	194.0 ± 54.4
GSH (g/L)	19.7 ± 7.1	19.3 ± 7.2
MDA equivalents (nmol/mL)	18.8 ± 6.5	16.4 ± 5.9

Data are mean ± standard deviation; BMI, body mass index; BMI = body weight (kg)/(body height (m))^2^; FRAP, ferric-reducing antioxidant power; GSH, reduced glutathione; GSH-Px, glutathione peroxidase; MDA, malondialdehyde; SOD, superoxide dismutase; WHR, waist to hip ratio; WHR = waist (cm)/hip (cm).

**Table 2:** Results of dietary survey ($\bar{x}$ ± s).

Parameter	Control	Brazilian Green Propolis
Energy (kcal)	1549.5 ± 423.4	1666.8 ± 589.0
Protein (g)	61.8 ± 18.7	62.8 ± 22.8
Lipids (g)	46.5 ± 25.0	57.2 ± 26.2
Carbohydrates (g)	226.6 ± 52.7	227.0 ± 81.3
Fiber (g)	13.0 ± 5.3	10.9 ± 3.8
Cholesterol (mg)	483.8 ± 227.8	470.5 ± 201.9
Retinol (μgRE)	516.7 ± 307.4	453.0 ± 189.5
Thiamin (mg)	0.8 ± 0.3	0.7 ± 0.3
Riboflavin (mg)	0.9 ± 0.4	0.8 ± 0.3
Niacin (mg)	11.5 ± 4.5	12.2 ± 5.6
Ascorbic acid (mg)	104.2 ± 60.7	76.8 ± 36.8 *
Tocopherol (mg)	15.3 ± 12.6	14.9 ± 7.4
Potassium (mg)	1933.6 ± 786.0	1769.1 ± 608.8
Sodium (mg)	943.8 ± 658.9	1309.5 ± 897.5
Calcium (mg)	569.2 ± 326.3	412.5 ± 184.8 *
Iron (mg)	20.7 ± 11.0	17.8 ± 6.4
Zinc (mg)	8.9 ± 2.6	9.2 ± 3.3
Selenium (μg)	50.7 ± 19.5	49.8 ± 18.4

Data are mean ± standard deviation; * *p* < 0.05, compared with the control group.

**Table 3:** Effects of Brazilian green propolis on glucose metabolism in T2DM patients.

Parameter	Control	Brazilian Green Propolis
Glucose (mmol/L)	8.4 ± 2.4	9.3 ± 2.6
Glycosylated hemoglobin (%)	7.6 ± 1.2	7.8 ± 1.3
Insulin (uIU/mL)	13.7 ± 4.1	11.9 ± 4.4
Aldose reductase (ng/mL)	0.62 ± 0.26	0.70 ± 0.23
Adiponectin (mg/L)	0.73 ± 0.14	0.69 ± 0.16

Data are mean ± standard deviation.

**Table 4:** Effects of Brazilian green propolis on antioxidant function and cytokines in T2DM patients.

Parameter	Control	Brazilian Green Propolis
FRAP (mmol/L)	0.71 ± 0.14	0.73 ± 0.08
SOD (U/mL)	102.8 ± 3.1	105.4 ± 2.9
GSH-Px (U/L)	259.9 ± 68.3	289.7 ± 87.7
GSH (g/L)	2.3 ± 0.9	6.3 ± 2.2 *
Total polyphenols (mmol/L)	199.9 ± 12.6	209.9 ± 16.9 *
MDA (mmol/L)	4.1 ± 1.1	4.0 ± 1.1
Ox-LDL (nmol/L)	2.8 ± 0.7	3.0 ± 0.8
Carbonyls (nmol/mg·prot)	0.59 ± 0.13	0.49 ± 0.13 *
LDH (U/L)	1446.7 ± 202.1	1329.1 ± 175.8 *
IL-1β (pg/mL)	18.7 ± 3.5	22.0 ± 4.5 *
IL-6 (pg/mL)	10.0 ± 5.0	18.1 ± 5.0 *
TNF-α (pg/mL)	20.7 ± 3.7	16.4 ± 9.1 *

Data are mean ± standard deviation; * *p* < 0.05, compared with the control group. FRAP, ferric-reducing antioxidant power; GSH, glutathione; GSH-Px, glutathione peroxidase; IL-1β, interleukin-1β; IL-6, interleukin-6; LDH, lactate dehydrogenase; MDA, malondialdehyde; Ox-LDL, oxidized low density lipoprotein; SOD, superoxide dismutase; TNF-α, tumor necrosis factor-α.

## Abbreviations

The following abbreviations are used in this manuscript:

DM	diabetes mellitus
T2DM	type 2 diabetes mellitus
BMI	body mass index
WHR	waist to hip ratio
ELISA	enzyme-linked immunosorbent assay
FRAP	ferric-reducing antioxidant power
SOD	superoxide dismutase
GSH-Px	glutathione peroxidase
GSH	glutathione
MDA	malondialdehyde
ox-LDL	oxidized low density lipoprotein
LDH	lactate dehydrogenase
IL-1β	interleukin-1β
IL-6	interleukin-6
TNF-α	tumor necrosis-factor α
ROS	reactive oxygen species
